# Predominance of IL-5-secreting drug-specific T cells in subgroups of patients with drug-induced delayed-type hypersensitivity reaction

**DOI:** 10.1186/2045-7022-4-S3-P35

**Published:** 2014-07-18

**Authors:** Wolfgang Pfützner, Carolin Intrup, Christian Möbs

**Affiliations:** 1 Department of Dermatology and Allergology, Germany; 2 Philipps University Marburg, Department of Dermatology and Allergology, Germany

## Background

Drug hypersensitivity reactions (DHRs) can be classified into IgE-induced, immediate type and T-cell mediated, delayed-type reactions. While the former are considered as Th2-driven immune responses characterized by IL-5-secretion, the later are regarded as mainly provoked by IFN-gamma-producing Th1 cells. However, it has been suggested that delayed-type DHRs could also be elicited by IL-5 or a mixed T cell cytokine pattern.

## Methods

We sought to characterize drug-specific T cell subpopulations in patients with delayed-type DHRs to beta-lactam antibiotics by enzyme-linked immunospot (ELISPOT) assay, a sensitive technique to detect antigen-specific cytokine-producing cells. Peripheral blood was drawn from eight patients who had experienced maculopapular exanthema as a clinical manifestation of a DHR against beta-lactam antibiotics (penicillin, amoxicillin or cefuroxim), which was confirmed by skin patch-test. The occurrence and frequencies of drug-specific IL-5-, IFN-gamma- and IL-10-producing T cells were analyzed by ELISPOT assay and compared to ten healthy controls without history of delayed-type DHRs.

## Results and conclusion

As expected, ELISPOT analysis of peripheral blood mononuclear cells (PBMC) revealed increases in drug-specific IFN-gamma-producing T cells. However, in a subgroup of patients with delayed-type DHRs, augmented numbers of drug-specific IL-5-secreting T cells were observed, regardless which drug was taken. Of note, IL-10 positive T cells were not decreased in drug-allergic patients when compared with healthy controls. Our results show that DHRs are characterized by distinct patterns of cytokine-secreting lymphocytes which resemble either Th1, Th2 or a mixed type of Th cells. Thus, determining both IFN-gamma- and IL-5-positive (but not IL-10-producing) cells by ELISPOT assay increases the sensitivity in detecting drug-specific T cells. This may help diagnosing drug-allergy in patients with suspected DHRs negative by other conventional allergy tests.

